# The Case of an 18‐Year‐Old Female With Persistent Vague Pain in the Top of the Occiput

**DOI:** 10.1002/acn3.70511

**Published:** 2026-08-08

**Authors:** Jianping Zhong, Binglin Lai

**Affiliations:** ^1^ Ganzhou Institute of Medical Imaging, Ganzhou Key Laboratory of Medical Imaging and Artificial Intelligence, Medical Imaging Center, Ganzhou People's Hospital, Ganzhou Hospital‐Nanfang Hospital Southern Medical University Ganzhou China

**Keywords:** bilateral thalamic lesions, cerebral venous thrombosis, iron deficiency anemia, straight sinus

## 1 Summary of Case (HPI, Relevant Exam Findings, and Relevant Data)

**FIGURE 1:**
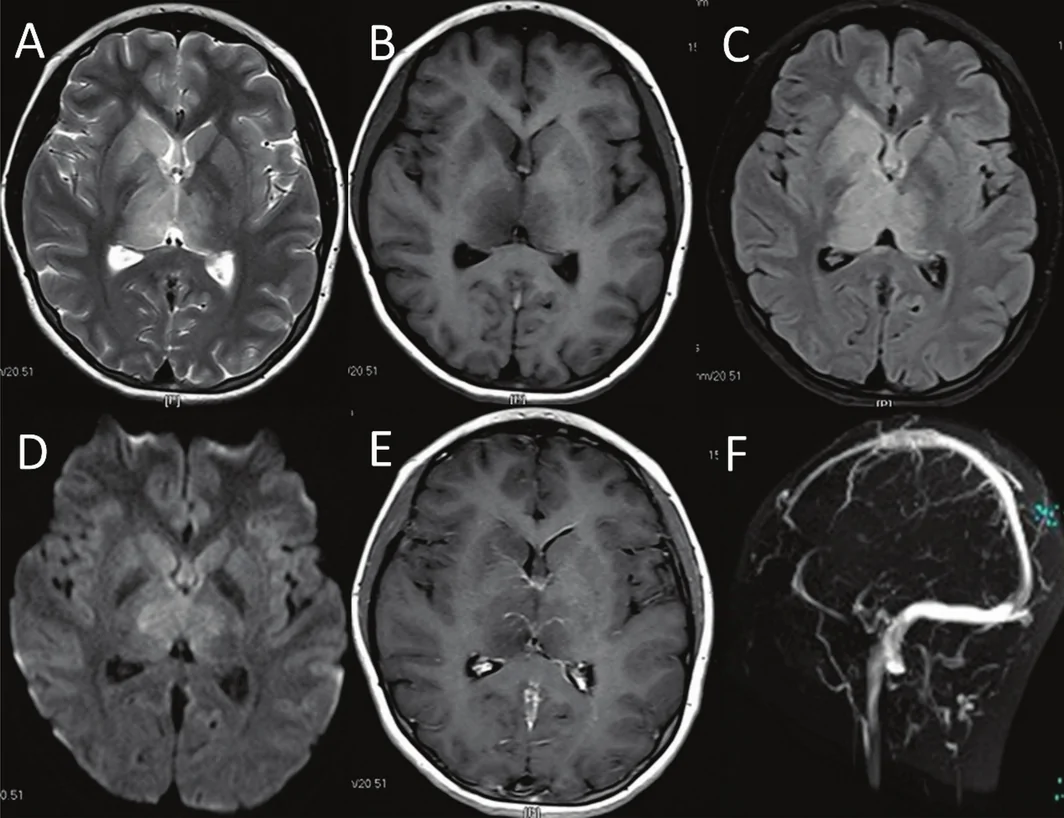
Hyperintense signals are observed in the bilateral thalami, right corpus callosum, and caudate nucleus on T2WI (A), FLAIR (C), and DWI sequences (D), with hypointense signals on T1WI (B). No contrast enhancement is seen on post‐contrast imaging (E). MRV shows nonvisualization of the straight sinus (F).

An 18‐year‐old female with no significant past medical history presented with a two‐year history of persistent dull pain localized to the occipital region. Each pain episode lasted approximately 3 to 5 s and resolved spontaneously without intervention. Over the 2 months prior to presentation, both the frequency and intensity of her headaches had markedly increased, prompting further evaluation. She denied associated symptoms including nausea, vomiting, visual disturbances, photophobia, phonophobia, focal weakness, numbness, or seizures. She had no history of oral contraceptive use, trauma, or prior hospitalizations. General and neurological examinations were normal except for mild conjunctival pallor. MRI of the brain demonstrated T2/FLAIR hyperintense signals in the bilateral thalami, corpus callosum, and caudate nucleus without contrast enhancement (Figure 1A–E). Laboratory studies revealed severe microcytic anemia (hemoglobin 61 g/L) and profoundly low ferritin (2.15 ng/mL). Lumbar puncture showed normal opening pressure (175 mmH_2_O) and normal CSF biochemistry. Plasma D‐dimer was within normal limits (0.41 mg/L). Magnetic resonance venography (MRV) showed nonvisualization of the straight sinus, confirming the diagnosis of deep cerebral venous thrombosis (CVT) (Figure 1F). The patient was treated with enoxaparin and iron dextran. Her headache improved within weeks. Follow‐up MRI with venography at 6 weeks demonstrated resolution of the thalamic lesions and recanalization of the straight sinus, and she remained asymptomatic on continued follow‐up.

## 2 Diagnosis

Deep Cerebral Venous Thrombosis (CVT) involving the straight sinus, likely secondary to iron deficiency anemia.

## 3 Take‐Home Points

- Iron deficiency anemia can predispose to cerebral venous thrombosis, even in young patients without traditional risk factors [1, 2, 3, 4, 5].
- Bilateral thalamic lesions on MRI should raise suspicion for deep cerebral venous thrombosis, particularly involving the straight sinus, even in the absence of confirmatory findings on initial tests like D‐dimer or CSF analysis.
- A normal D‐dimer does not exclude the diagnosis of cerebral venous thrombosis, particularly in subacute or chronic cases.
- Headache in the setting of anemia warrants evaluation for both hypoxic and thrombotic causes.

## Author Contributions

J.Z. contributed to the conception, the acquisition, the literature search and preparing the figures; B.L. contributed to drafting the text and approved the manuscript.

## Funding

The authors have nothing to report.

## Conflicts of Interest

The authors declare no conflicts of interest.

## Data Availability

The data that support the findings of this study are available on request from the corresponding author. The data are not publicly available due to privacy or ethical restrictions.
